# Stomatal opening efficiency is controlled by cell wall organization in *Arabidopsis thaliana*

**DOI:** 10.1093/pnasnexus/pgad294

**Published:** 2023-09-11

**Authors:** Sedighe Keynia, Leila Jaafar, You Zhou, Charles T Anderson, Joseph A Turner

**Affiliations:** Department of Mechanical and Materials Engineering, University of Nebraska-Lincoln, Lincoln, NE 68588, USA; Department of Biology and Intercollege Graduate Degree Program in Molecular Cellular and Integrative Biosciences, Pennsylvania State University, University Park, PA 16802, USA; Center for Biotechnology, University of Nebraska-Lincoln, Lincoln, NE 68588, USA; Department of Biology and Intercollege Graduate Degree Program in Molecular Cellular and Integrative Biosciences, Pennsylvania State University, University Park, PA 16802, USA; Department of Mechanical and Materials Engineering, University of Nebraska-Lincoln, Lincoln, NE 68588, USA

## Abstract

Stomatal function in plants is regulated by the nanoscale architecture of the cell wall and turgor pressure, which together control stomatal pore size to facilitate gas exchange and photosynthesis. The mechanical properties of the cell wall and cell geometry are critical determinants of stomatal dynamics. However, the specific biomechanical functions of wall constituents, for example, cellulose and pectins, and their impact on the work required to open or close the stomatal pore are unclear. Here, we use nanoindentation in normal and lateral directions, computational modeling, and microscopic imaging of cells from the model plant *Arabidopsis thaliana* to investigate the precise influences of wall architecture and turgor pressure on stomatal biomechanics. This approach allows us to quantify and compare the unique anisotropic properties of guard cells with normal composition, lower cellulose content, or alterations in pectin molecular weight. Using these data to calculate the work required to open the stomata reveals that the wild type, with a circumferential-to-longitudinal modulus ratio of 3:1, is the most energy-efficient of those studied. In addition, the tested genotypes displayed similar changes in their pore size despite large differences in wall thickness and biomechanical properties. These findings imply that homeostasis in stomatal function is maintained in the face of varying wall compositions and biomechanics by tuning wall thickness.

**Significance Statement**

Guard cells are critical for photosynthesis, and their cell wall composition and geometry control their response to turgor pressure. Mechanisms underlying this process are poorly understood with respect to the energy efficiency of pore opening. Here, we quantify the anisotropic mechanical properties and turgor pressure of guard cells using nanoindentation measurements in three orthogonal directions to corroborate an associated computational model of the experiments. The model is then used to determine the stomatal opening efficiency for several genotypes with cellulose or pectin modifications. The wild type is shown to be most efficient due to its high anisotropy and thickness distribution. The mutants have wall thickness differences which may be a compensation to achieve normal stomatal response despite wall composition deviations.

## Introduction

Stomatal guard cells in plants play a vital role in photosynthesis because they control the dynamics of stomatal pores, openings in the leaf epidermal surface. The ability of guard cells to regulate pore width efficiently is critical to the exchange of CO_2_ and water vapor which sustains plant life (1). Dysfunctional stomatal complexes can restrict CO_2_ access or cause excessive water loss if the pore aperture is too small or too large, respectively, relative to its optimal size. Guard cell dynamics are driven by environmental or intrinsic stimuli that result in ion and water flux across the plasma membrane (2). However, overall stomatal response is a complex interaction between turgor pressure, the material organization that controls the mechanical properties of the guard cell wall, boundary conditions imposed by neighboring epidermal cells (3), and overall cell geometry including cell shape and wall thickness. Researchers are still discovering the genetic and molecular control mechanisms for each of these features. Thus, a clear understanding of these biomechanical interactions is needed to develop new crop varieties that are more efficient at capturing CO_2_ and more resistant to drought (4), an important global need due to the changing climate.

Current microscopy techniques allow guard cell geometry to be measured accurately, but the role of subcellular components in stomatal function is more challenging, and many questions remain (5–8). Several efforts have been made to correlate cytoskeleton components with guard cell function (9, 10), and recent research has begun to examine the mechanical properties of the cell wall (11–13). Such information is critical if robust computational models of guard cell dynamics are to be developed to interpret changes that result from genetic engineering. The guard cell wall is a composite of stiff cellulose microfibrils (CMFs) embedded within a more compliant matrix that includes pectin, hemicelluloses, and structural proteins. In eudicots, the CMFs wrap around the circumference of each guard cell (14, 15), giving the wall an anisotropic material response which influences stomatal opening and closing behavior.

Changes in the wall structure of guard cells can affect their function (11, 12, 14, 16–21): changes in CMF content affect the degree of anisotropy as well as cell wall stiffness and pore width (17, 22), and cellulose has been observed to unbundle and bundle during stomatal opening and closure, respectively (16). In *A. thaliana*, the *CELLULOSE SYNTHASE* (*CESA*) gene family has 10 members (23). Among those, *CESA3* is one isoform that is involved in primary wall biosynthesis (24–26). Specifically, for primary wall-forming cellulose synthase complexes (CSCs), biochemical and genetic studies indicate that *CESA3* is an essential component of the CSC (27, 28). Stomata in a *cesa3^je5^* mutant with cellulose deficiency (27, 28) have been reported to open wider and have a thicker cell wall at full maturity (14, 16, 17). Although cellulose content is significantly reduced in this mutant, the cell wall was estimated to be stiffer and more anisotropic than wild type (WT) based on computational models that derived wall mechanical properties from dynamic cell geometries (18). These results were interpreted as reflecting defects in cellulose bundling and/or cross-linking by matrix polysaccharides, while in Rui et al. (16), model mutants with deficiency in cellulose were modeled with a more compliant cell wall that opened wider. The cell wall determinants of stomatal behavior are not limited to CMFs and their orientation alone. The composition of the wall matrix and its constituents of pectins and hemicelluloses, including homogalacturonan (HG) and xyloglucan, respectively (10–12, 17, 18, 29–35), is important for stomatal function as are the interactions between any two components, such as hemicellulose interactions with CMFs (35, 36).

Among matrix components, pectins have a significant role in eudicot stomatal function (12, 17, 18, 30, 33). One aspect has been highlighted with respect to pectin-based polar stiffening which restricts the stomatal complex (13). The importance of pectins and their effects on the mechanical properties of guard cells has been demonstrated in mutants with altered pectin properties, such as those for *PECTATE LYASE LIKE12* (*PLL12*) (11), which is required for normal stomatal function. *POLYGALACTURONASE INVOLVED IN EXPANSION3* (*PGX3*) (37) is another gene whose product modulates pectin size and abundance and maintains proper opening and closing dynamics of mature stomata. An *Arabidopsis* line that overexpresses *POLYGALACTURONASE INVOLVED IN EXPANSION1* (*PGX1-OE*) has pectic HG with a smaller average molecular mass (38). Existing computational models (17) built on geometrical measurements alone (14, 16, 17) suggest that the walls of *PGX1-OE* guard cells have a lower elastic modulus in the longitudinal direction but a higher modulus in the circumferential and radial directions that were assumed equal. Atomic force microscopy (AFM) results (39) suggest that for some cell types pectin demethylesterification decreases cell wall stiffness and reduces growth anisotropy.

Direct mechanical measurements of guard cells are challenging because wall deformation behavior is coupled with changes in turgor pressure. Nanoindentation, also called instrumented indentation testing (IIT), was recently used to measure the force–displacement behavior of guard cell walls in the normal direction, i.e. perpendicular to the leaf surface (11). Wall modulus and turgor pressure were separated using a computational model that used the measured cell geometry and an assumed material anisotropy for the wall. An iteration procedure was used to quantify wall modulus and turgor pressure so that the model matched the experimental nanoindentation data. Those results revealed dynamic changes in wall modulus and turgor pressure during light-induced stomatal opening and dark-induced stomatal closure, tracking an initial jump in wall modulus followed by a steady decline in modulus during a 60 min light stimulus and an initial jump in turgor pressure followed by a steady increase over 60 min. This approach worked well, but the inherent in-plane anisotropy of the guard cell wall could not be measured because the applied load was perpendicular to the material symmetry plane of the wall.

Here, we performed nanoindentation measurements to capture the mechanical response of guard cell walls in three orthogonal directions. Finite element (FE) models defined from measured cell geometry were used to quantify turgor pressure and wall modulus, including the in-plane anisotropy. This approach was used to study mutants that affect cellulose or pectin, and changes in cell wall properties were quantified. The model was then used to examine stomatal opening efficiency for the selected genotypes.

## Results

### Three-dimensional nanoindentation reveals anisotropic wall stiffness in guard cells

**Fig. 1.**
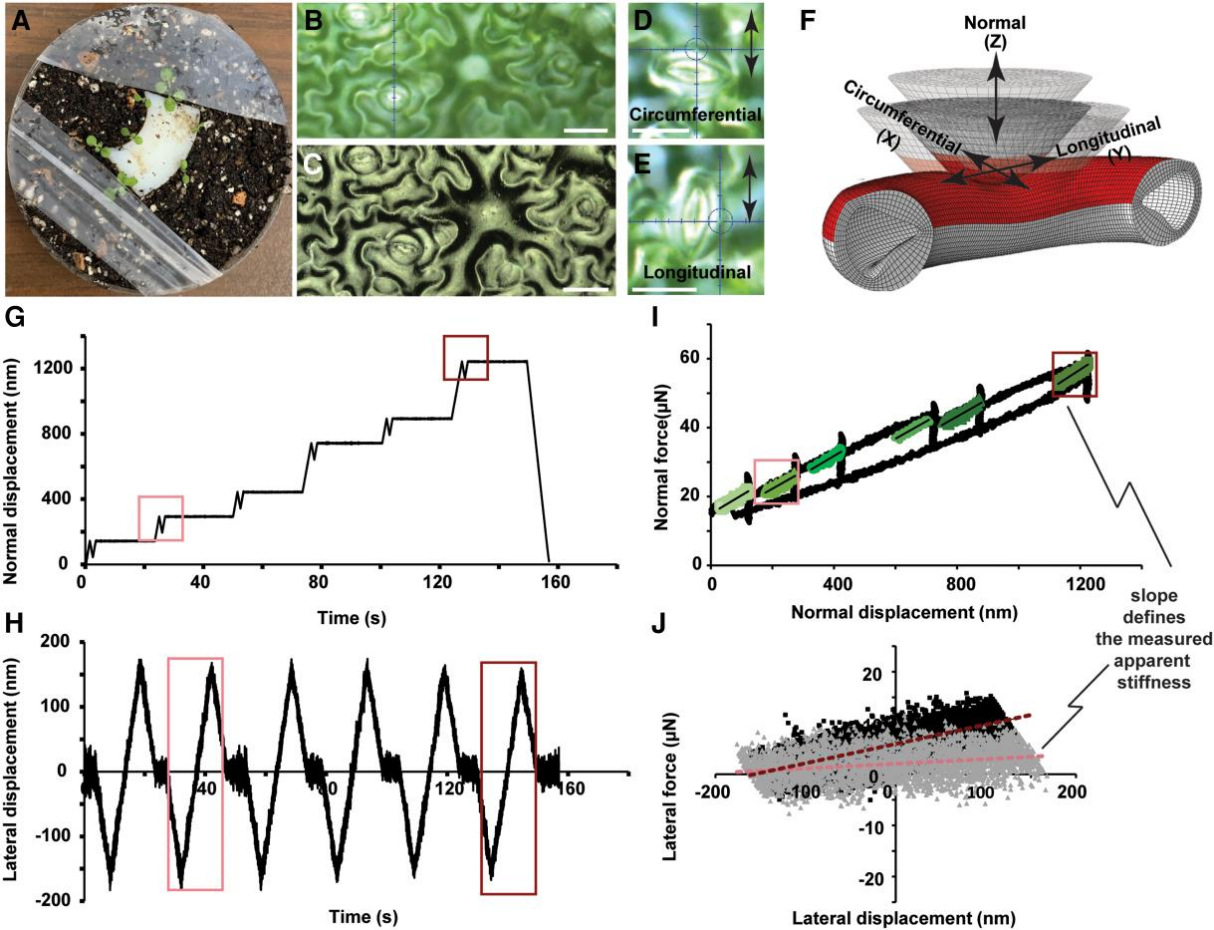
Multidimensional nanoindentation measurements of normal and lateral stiffness of the cell wall. A) The abaxial side of a cotyledon was glued to a holding plate that provided a fixed sample for nanoindentation measurements on the adaxial side. B) High magnification images from the 50× lens mounted to the nanoindenter enabled us to indent on the targeted location of the cell with high accuracy. C) The 3D geometry of the cell was measured accurately using the laser microscope. Scale bars in (B) and (C) are 20 µm. D) Measurements of circumferential lateral indentation and longitudinal lateral indentation were made by rotating the sample 90° as shown in (E). The arrows show the direction of tip lateral motion. F) Cell wall nanoindentation in three orthogonal directions: circumferential (X), longitudinal (Y), and normal (Z) defined in the FE model based on the nanoindentation measurements. G and H) Input normal and lateral displacement profiles used for the measurements. After each normal indentation (loading and unloading) at a specific depth, the tip moved laterally to quantify the lateral stiffness in the longitudinal or circumferential direction depending on cell orientation. Measurement depths of 300 and 1,250 nm were used for the iterative FE analysis. I and J) The slopes of unloading sections of measured force vs displacement curves for normal and lateral directions shows the stiffness used for the analysis. The shallow and deep normal stiffness is shown with light and dark boxes in (I). The shallow and deep lateral stiffnesses were measured by the slope of the two linear fits shown in (J).

Nanoindentation experiments in three orthogonal directions (normal and two lateral) were performed on mature guard cells of *Arabidopsis* cotyledons with open stomata to capture the three-dimensional (3D) anisotropic mechanical properties of the cell wall. We studied the response of WT (Col-0) stomata as well as mutants with cellulose deficiency (*cesa3^je5^*) or putative lower (*PGX1-OE* and *PGX3-OE*) or higher (*pgx3-1*) pectin molecular weight (14, 38). Cotyledons were mounted on a support to expose the adaxial side for the experiments (Fig. 1A). Then, a 50× objective within the nanoindenter was used to identify guard cells for the measurements (Fig. 1B). A laser scanning confocal microscope was used after nanoindentation to capture the geometry of the same guard cells of interest (Fig. 1C). The measurement data provided accurate geometries of each guard cell for computational models of the experiments. Our goal was to measure the lateral response in two orthogonal directions (longitudinal and circumferential), but the transducer was limited to displacement in the normal direction (i.e. perpendicular to the plane of the leaf) and a single lateral direction. Therefore, lateral indentations were performed in one direction and then the sample was rotated 90° and measurements were repeated in the perpendicular direction. This approach allowed the measurements to capture mechanical response with respect to the circumferential direction (Fig. 1D) and the longitudinal direction (Fig. 1E). Figure 1F shows the three measurement directions, with respect to the guard cell for normal and lateral stiffness measurements. Example normal and lateral displacement profiles as a function of time are shown in Fig. 1G and H, respectively. For each direction, unloading segments were used to quantify stiffness (i.e. the slope of the force–displacement curve) at six specific depths (11). After engaging with the cell wall, the nanoindenter tip indented the cell in the normal direction to each depth increment after which an unloading–reloading cycle of 100 nm was performed. At the end of each normal direction measurement step, the normal displacement was held constant and a lateral indentation step was initiated. A lateral tip displacement of ±150 nm was then used, and the lateral force was measured. After each lateral step, the normal displacement was increased to the next depth increment and the process was repeated. Example force–displacement curves for normal and lateral indentations are shown in Fig. 1I and J, respectively. Stiffnesses at each specific depth and direction were quantified from the unloading force–displacement response. The experimental stiffness results from measurements on nine guard cells for WT and nine for each mutant are provided in Figs. S1 and S2; in general, normal stiffness was lower in all mutant genotypes than in WT guard cells, with lateral stiffness showing more variability. The normal stiffness values increase with depth, in part due to the role of turgor pressure but also because of wall thickness and cell geometry. Thus, a computational model was used to separate the turgor pressure information from the wall deformation response.

### Computational models to determine wall modulus values from measured cell stiffness values

Measured values of cell stiffness are influenced by both turgor pressure and the mechanical properties of the cell wall (i.e. wall modulus in the radial, circumferential, and longitudinal directions) as well as geometry (wall thickness and cell size). Thus, a robust computational model is needed to determine values of wall modulus and turgor pressure that match the raw nanoindentation data. Computational models of plant cells are increasingly used to understand their biological attributes and responses (17, 40–49). In our case, such models require several initial pieces of information: (i) a wall thickness profile, (ii) the geometry of each guard cell, and (iii) contact properties between the nanoindenter tip and cell wall.

**Fig. 2.**
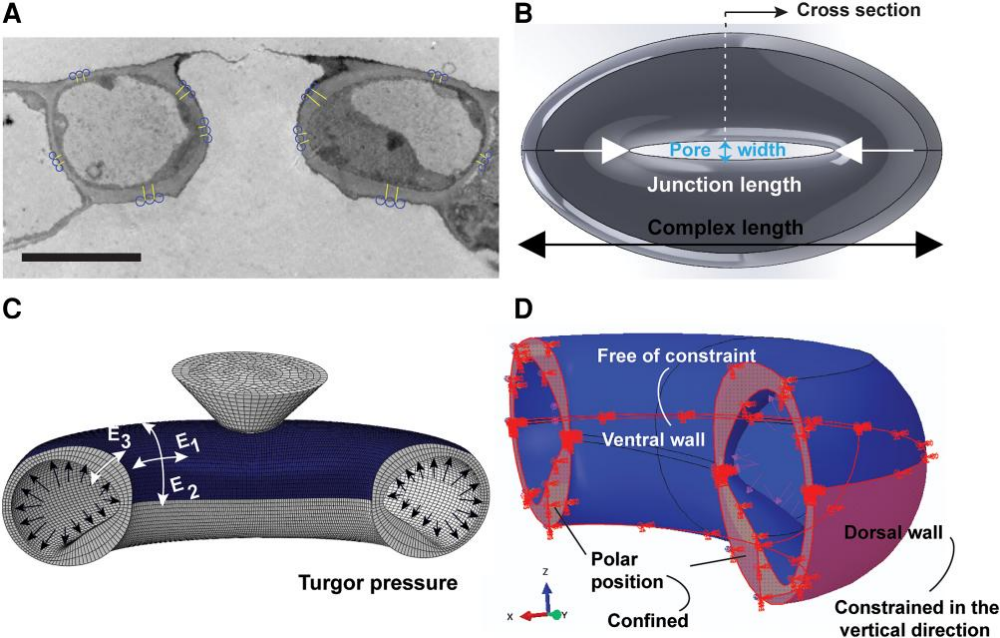
A robust FE model requires accurate structural geometry. A) TEM image of the guard cell cross-section of a WT Col-0. Five different regions of the guard cell were used to build the FE model cross-section (scale bar is 5 µm; image resolution is 3.1 nm/pixel). The geometry of the guard cells was built in SolidWorks (B) based on the TEM images and dimensions from microscopy. C) The geometry of the cells and scanned nanoindenter conical tip were imported into Abaqus for FE analysis. D) Boundary conditions were assigned to the end of stomata and dorsal region to represent the effect of neighboring cells and the partner guard cell, respectively.

We created several FE models for representative cells of each genotype that were indented (e.g. Fig. 1). The models were created based on specific geometric microscopy data for each cell whereas the wall thickness profile for each genotype was measured from transmission electron microscopy (TEM) images of guard cell cross-sections (Fig. 2A; Fig. S3 and Table S1). Microscopic images (e.g. Fig. 1) were used to quantify the pore width, complex length, and junction length of each stomatal complex to create the models as defined in Fig. 2B. The cell wall was modeled as an anisotropic viscoelastic material with longitudinal (*E*_1_), circumferential (*E*_2_), and radial (*E*_3_) moduli (Fig. 2C). CMFs are synthesized circumferentially in guard cells (14, 15) such that the modulus of the composite wall was assumed to have the same values in directions perpendicular to the cellulose fibers (i.e. transverse isotropy). Thus, we assumed that *E*_1_ = *E*_3_ for all models. Figure 2C shows an example FE model of a Col-0 guard cell. Note that the models reflected differences in wall thickness and geometry between genotypes (see Table S1). Viscoelastic properties were defined from literature values (40). The FE model of the indenter tip was based on a confocal scan of the actual tip used for the nanoindentation experiments. The boundary conditions defined for the model are shown in Fig. 2D. We assumed that the polar positions of the guard cells were confined, the ventral edges were free of constraint, and the dorsal edges were constrained in the vertical (anticlinal) direction to represent constraints from adjacent pavement cells. Each FE model was used iteratively to match the measured values of stiffness (Figs. S1 and S2) as a function of indentation depth and direction. The flowchart of the process used to calculate the wall moduli, turgor pressure, and geometrical deformation (pore width) to match the experimental results is provided in Fig. S4. The inversion process was based on measurements at depths of 300 and 1,250 nm because these limits represent the sensitivity range of wall modulus and turgor pressure on the measured stiffness: shallow indentations are mostly affected by cell wall properties, whereas the deep indentations are influenced more by turgor pressure. Shallow lateral stiffness measurements were more sensitive to the moduli in the circumferential and longitudinal directions. The ratio of these moduli was then used for comparison with the measured normal stiffness. Then, the turgor pressure was matched based on the deep normal stiffness measurements. Finally, after the wall moduli and turgor pressure were estimated, the measured change in pore width was compared with the model result. If the change was not consistent with the measurements, the iterative cycle was repeated for new values of wall moduli and turgor pressure. After convergence, the process provided estimates for wall moduli and turgor pressure for a given nanoindentation measurement. This pipeline provided simultaneous wall modulus values, in three dimensions, plus turgor pressure, for physiologically active guard cells.

### Testing the effects of tip-sample contact on lateral indentation measurements

The contact properties (friction, adhesion, etc.) between the tip and plant surface have little influence on the indentation measurements in the normal direction (50), so the contact properties were not examined previously (11). For lateral stiffness measurements, the contact is critical because sliding would influence the interpretation of the measurements for which the goal is to assess the lateral material behavior. For this reason, the contact properties were quantified using a series of lateral measurements with variable displacement from 300 to 4 µm. The point at which sliding initiated (∼250 to 300 nm) could be easily assessed from lateral force vs. lateral displacement data (Fig. S5C). The lateral indentation measurements used to determine wall modulus were limited to ±150 nm such that no sliding was expected for assessment of the moduli. The sliding initiation point and the subsequent slope during sliding were used to define the contact parameters of the FE model including the friction coefficient, the shear stress limit, and the fraction of characteristic surface dimension (Fig. S5E to G). To ensure that lateral indentation did not damage the leaf surface, measurements were repeated and the results showed no difference in outcome (Fig. S5D).

### Cellulose increases wall anisotropy for normal stomatal function

**Fig. 3.**
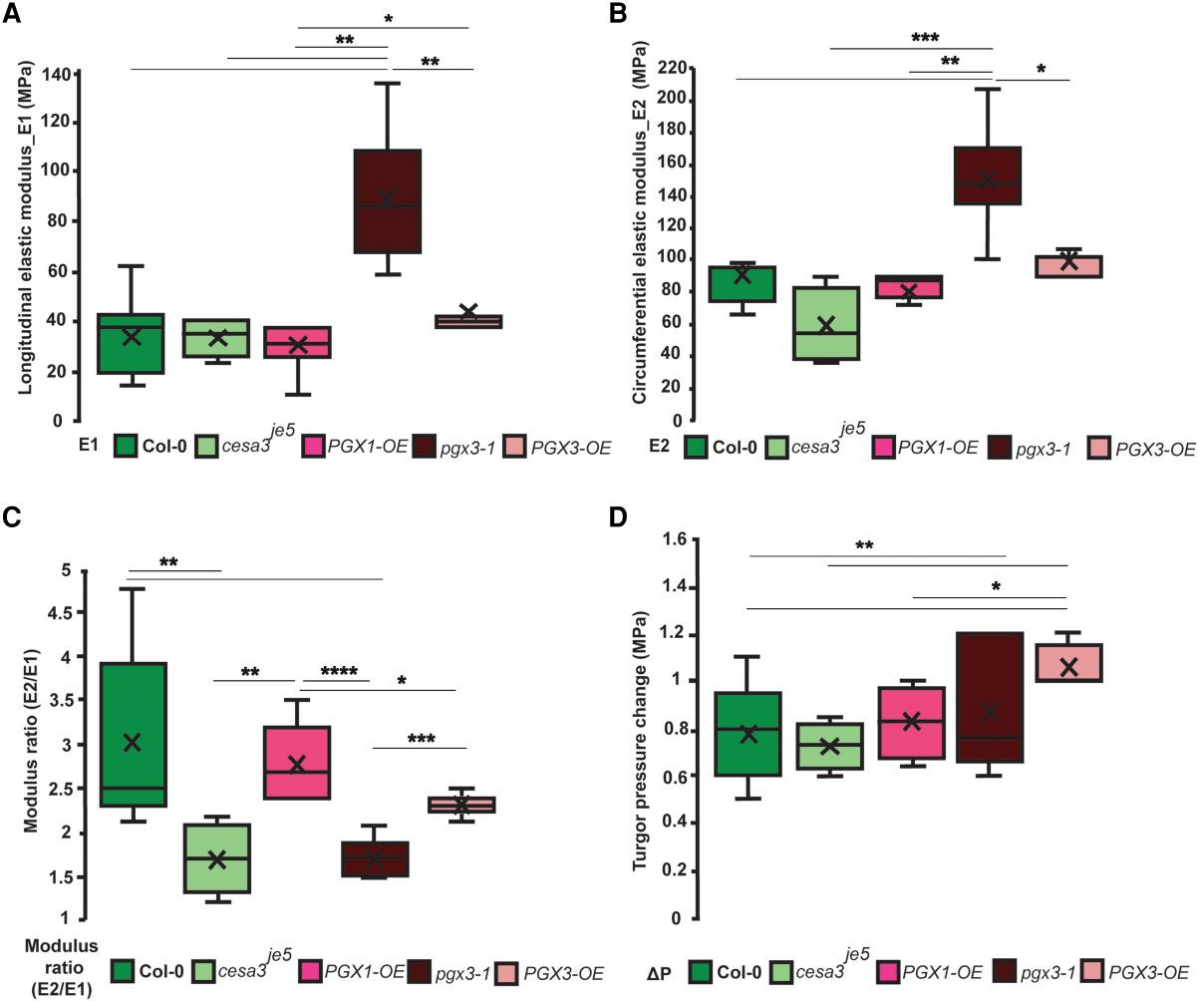
Experimental–computational results of mutant guard cells reveal significant differences between their anisotropic ratio and wall modulus. For each genotype, cells were selected for the analysis (see Table S2 for all results). (A) Circumferential and (B) longitudinal elastic moduli in WT and four mutants with cellulose defect (*cesa3^je5^*) and altered pectin structure (*PGX1-OE*, *pgx3-1*, and *PGX3-OE*). C) Anisotropy ratio of each guard cell is calculated by the ratio of circumferential-to-longitudinal elastic modulus. D) Turgor pressure change for opening shows significance from WT for *pgx3-1* and *PGX3-OE*.

We next analyzed the functions of different cell wall components in pore opening using several mutants. We first examined the *cesa3^je5^* mutant that is cellulose deficient (14). In adult leaves of *cesa3^je5^*, stomatal complexes exhibit geometric differences compared with Col-0 stomata, such as larger apertures in the closed and open states, whereas pore length is smaller than WT (14), resulting in similar pore area at the open state. Hence, *cesa3^je5^* FE models require major geometric changes including cell wall thickness, which is another key difference of this mutant (Fig. S3B). Similar to published data gathered from cross-sections stained with toluidine blue reported by Yi et al. (17), our TEM images of cross-sections of *cesa3^je5^* guard cells showed thicker walls at different positions around the cells relative to Col-0 (Fig. S3B and Table S1). The FE models of *cesa3^je5^* were used iteratively to match stiffness measurements from nanoindentation (Figs. S1D to F and S2). The results (Fig. 3A to D) showed that the walls of *cesa3^je5^* guard cells have a slightly lower circumferential modulus than Col-0 guard cells and are much less anisotropic. No significant difference in the turgor pressure required for opening was observed despite differences in the geometrical features and cell wall properties of the cellulose-deficient mutant. These results imply that lower cellulose content in *cesa3^je5^* stomatal complexes significantly reduces wall anisotropy. The thicker cell wall appears to compensate in part for the modulus changes such that the change in pore area is not affected significantly by the altered wall properties relative to Col-0. The anisotropy is a qualitative indication of the cellulose-to-pectin volumetric ratio because the alignment of cellulose within guard cells is fairly well understood and the wall matrix is thought to be isotropic (15, 16).

### Pectin molecular mass affects wall anisotropy and stiffness

Pectins have been implicated in stomatal biomechanics and are hypothesized to influence both polar stiffening (13) and the kinetics of stomatal opening (37). The effect of pectin-related genes in guard cell function has been investigated using a set of mutants that display alterations in total polygalacturonase activity, pectin molecular mass, and wall composition (17, 37, 38). A subset of these plants either overexpress or are deficient in *POLYGALACTURONASE INVOLVED IN EXPANSION1* (*PGX1*) or *PGX3*, and we tested those here.

The first mutant with altered pectin structure we analyzed was *PGX1-OE*, which overexpresses *PGX1*. *PGX1-OE* leads to the degradation of HG, creating pectin chains of smaller molecular weight (38). *PGX1-OE* stomata exhibit an increase in stomatal apertures in the closed state (17). Nanoindentation and TEM measurements of *PGX1-OE* guard cells showed that this mutant has lower normal and circumferential stiffness (Figs. S1 and S2) and a thinner cell wall (Fig. S3C and Table S1). The computational inversion of the experimental data shows no significant change in cell wall modulus, degree of anisotropy, or turgor pressure change required for pore opening in comparison with Col-0 (Fig. 3D). These results are consistent with previous models based on geometric measurements of pore opening alone (17) in terms of the reduction of wall modulus in the longitudinal direction.

We tested the influence of another polygalacturonase gene (*PGX3*) product on guard cell wall stiffness. The *PGX3* knockout mutant (*pgx3-1*) has higher molecular weight pectin, whereas the overexpression of *PGX3* (*PGX3-OE*) creates low molecular weight pectin which changes stomatal dynamics (37). The material properties of *pgx3-1* and *PGX3-OE* guard cells, based on the 3D nanoindentation and FE models, showed differing trends relative to Col-0 (Fig. 3A to D). Inversion of the experimental data using the computational model for *pgx3-1* revealed that wall modulus was significantly larger in both the circumferential and longitudinal directions (Fig. 3A and B), while the anisotropy ratio *E*_2_/*E*_1_ was much lower (Fig. 3C). In addition, there was a reduction in wall thickness in comparison with Col-0 that might reflect the smaller sizes of stomatal complexes in the knockout (37). When these properties were used in the analysis of pore width (Fig. S2), the impact on stomatal opening was minor, as had been observed for *pgx3-1* stomata in adult leaves (37). In contrast to the knockout, *PGX3-OE* showed a reduction in wall thickness in comparison with Col-0 but no significant changes in the longitudinal or circumferential moduli or cell wall anisotropy. Similar to the results for *PGX1-OE*, the only significant difference was the change in turgor pressure.

### Cell wall organization optimizes guard cell efficiency

The moduli of the cell wall can be examined within the context of a fiber–matrix composite to provide further insights into material changes observed within the walls of living plant cells. This analysis is based on a cell wall composed of fibers, here CMFs with modulus *E*_f_ embedded within a matrix with modulus *E*_m_. For guard cells, the alignment of the fibers is in the circumferential direction. In this case, we assume that the elastic modulus in the longitudinal direction (i.e. perpendicular to the fibers) is solely due to the matrix such that *E*_1_ = *E*_m_. The modulus in the circumferential direction is then


(1) $$E_{2}=V_{m}E_{m}+\;V_{f}E_{f}=(1-V_{f})E_{m}+\;V_{f}E_{f},$$

where *V*_m_ and *V*_f_ define the volume fractions of the matrix and fibers, respectively, with the constraint that *V*_m_ + *V*_f_ = 1. The anisotropy ratio of the wall is then given by


(2) $$\frac{E_{2}}{E_{1}}=(1-V_{f})+\;V_{f}\;\frac{E_{f}}{E_{m}}=1-V_{f}\left(1-\;\frac{E_{f}}{E_{m}}\right).$$

Our combined measurements and FE model provide values for *E*_1_ = *E*_m_ and *E*_2_ such that the modulus of the fibers can be estimated if their volume fraction is known. Thus, assuming a volume of fibers from 15 to 40% (51–55), we find 342.6 MPa > *E*_f_ > 107.4 MPa for Col-0 (note that a lower percentage of CMFs requires a larger fiber modulus *E*_f_). This range is less than that of pure crystalline cellulose as expected. If we assume the same range of *E*_f_ for the mutants, we can estimate the volume fraction of their CMFs as shown in Table 1. It is known that cellulose content and thus presumably the density of CMFs in *cesa3^je5^* are reduced (14). This analysis provides a quantitative estimate of this reduction within the wall of the living cell that is on the order of a factor of ∼1.25–1.97 which is consistent with cellulose content measurements (14). The changes in predicted fiber volume fraction for the PGX mutants might be related to increases in matrix production, specifically pectin, or changes in the pectin molecular mass, degree of cross-linking, and/or turnover rate in the wall. The estimate of *V*_f_ increases for *PGX1-OE* and *PGX3-OE* mutants relative to Col-0. If the volume fraction of CMFs is fixed for *PGX1-OE* relative to Col-0, the reduction in wall moduli suggests that the pectin of *PGX1-OE* has a reduced stiffness. This change may be a direct result of the reduced molecular mass of the pectic HG. This approach to estimate wall composition breaks down for *pgx3-1* which has a much higher stiffness in both longitudinal and circumferential directions. Its behavior shows that the molecular weight of pectin is important for overall cell stiffness and anisotropy.

**Table 1.** Estimated volume fraction of CMFs in guard cell walls for select mutants.

Mutant	Minimum	Maximum
Col-0	15.0%	40.0%
*cesa3^je5^*	7.6%	31.7%
*PGX1-OE*	17.0%	68.4%
*pgx3-1*	—	—
*PGX3-OE*	18.7%	87.4%

**Fig. 4.**
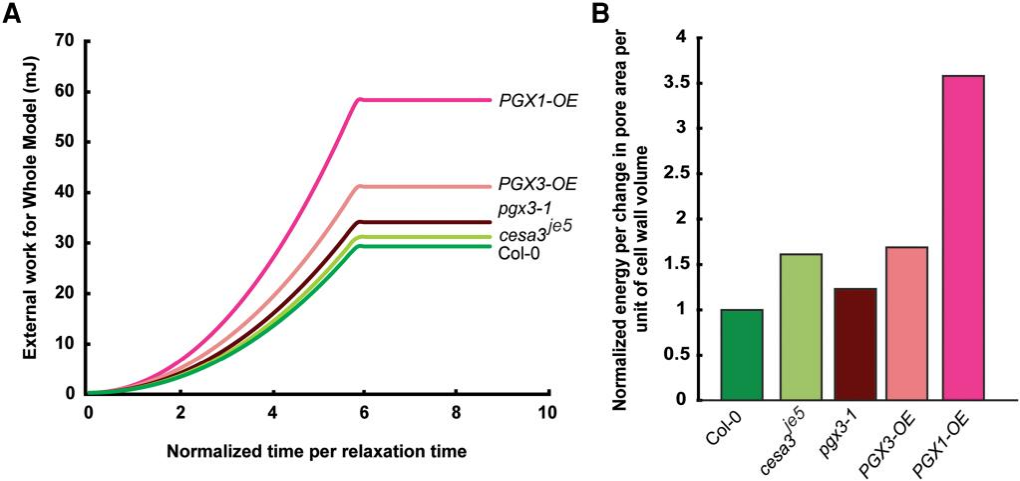
WT cell wall structure and composition are the most efficient for stomatal opening. A) Energy to open the pore calculated from each model based on the measured geometry and mechanical properties. B) Energy normalized by change in pore area and total cell wall volume to represent opening efficiency relative to the amount of material required to create the wall. The value for Col-0 is set to unity for comparison.

Despite differences in wall properties and cell geometry, the stomatal complexes of WT and the tested mutants all function effectively. However, not all wall compositions are equally efficient with respect to guard cell opening. Figure 4A shows the work as a function of time during simulated guard cell opening for each genotype based on the genotype-specific FE models and wall properties. The change in turgor pressure for these simulations occurred for a time that was 10 times the viscoelastic time constant assumed for the wall material model (11). The opening energy per change in pore area and cell volume (Fig. 4B) shows that the wall organization for Col-0 is the most efficient by a wide margin, especially in comparison with *PGX* overexpression lines. In addition, genotypes with thicker walls would have an additional energy penalty in terms of the energetic requirements for the synthesis and trafficking of additional wall polymers, which implies that the overall efficiency of Col-0 may be even better. Further research is required to quantify the efficiency of pore opening more precisely, but this value is likely a critical determinant of energetic efficiency for stomatal complexes, which must open and close frequently and repeatedly over the lifetime of a leaf or plant to optimize photosynthesis and water transport.

## Discussion

Our approach of 3D nanoindentation combined with geometrically accurate FE modeling was able to quantify differences in cell wall properties of Col-0 stomatal guard cells and those with differing cellulose and pectin composition and demonstrated the impacts of these changes on the efficiency of guard cell function. The ability to quantify in-plane anisotropy provides a unique view of the organization of wall constituents manipulated by genetics and other factors. Mechanical measurements on cell walls are crucial to validate and test FE models, and the three-dimensional measurements described here can make valuable contributions toward this end.

The analysis of mutants with altered wall composition suggests that the cellulose-deficient mutant (*cesa3^je5^*) exhibits significant reduction in wall anisotropy. The related changes in material stiffness might require the cell to produce a thicker wall to achieve sufficient pore size during opening; this compensation might occur via wall integrity sensing (56). The analysis here also provides a quantitative estimate of the decrease in the fraction of the wall occupied by CMFs in the cellulose-deficient mutant. These results are consistent with observations by Rui et al. (14) but differ from the assumptions used for a computational model of *cesa3^je5^* guard cells (17) that was based on pore opening alone and assumed equal circumferential and radial moduli.

The results from mutants with altered pectin structure show that pectic HG with a smaller average molecular weight greatly reduces the opening efficiency of the guard cell. *PGX1-OE* has pectin with lower molecular mass than Col-0, but its change in pore area during opening is very similar. Statistically significant changes were not observed in *PGX1-OE* or *PGX3-OE* for wall modulus in either direction or wall anisotropy. Wall thickness changes were clear, and the overall opening efficiencies were the worst of those studied. The mutant with higher molecular weight pectic HG had much stiffer walls and much lower anisotropy, showing that pectin is important for wall integrity and performance. Previous AFM measurements (13, 39, 57) showed a correlation between the degree of pectin methylesterification and wall stiffness in guard cells and other tissues. In addition, these results support observations by Rui et al., suggesting that *PGX3* controls stomatal dynamics by tuning pectin size and abundance (37).

Although the wall mechanical properties for each genotype studied here differ, only small differences were observed in the change in pore area when the cells were exposed to light. This result suggests that the plant regulates pore opening even under conditions of altered wall composition by building an appropriately thicker or thinner wall to maintain homeostasis. For instance, when the cell wall materials contributing to cell architecture are less stiff, a thicker wall might be needed to support effective environmental responses and gas transport. This outcome suggests that stomatal opening is conserved across varying genotypes or ecotypes. Future ecophysiological research could explore this hypothesis more fully.

## Materials and methods

### Experimental design

The goal of this study was to explain differences in the function of guard cells (Col-0 and select mutants) while limiting assumptions about wall properties. The combination of normal and lateral nanoindentation measurements, interpreted using a computational model, provided the basis for the conclusions. Based on this approach, we were able to analyze guard cell function and interpret the role of specific genes and wall components accordingly.

### Plant growth conditions

For biological assays, *Arabidopsis thaliana* Col-0 ecotype seeds expressing a plasma membrane marker, LTI6b-GFP (58), were sterilized in 30% bleach + 0.1% sodium dodecyl sulfate (SDS) for 20 min and then stratified at 4°C for 3–10 days before being plated on Murashige and Skoog (MS) plates containing 2.2 g/L MS salts (Caisson Laboratories), 0.6 g/L 2-(N-morpholino)ethanesulfonic acid (MES), 1% (*w*/*v*) sucrose, and 0.8% (*w*/*v*) agar (Sigma), pH 5.6. Seedlings were grown at 22°C under 8 h or 24 h of illumination at ∼800 photosynthetic photon flux density (PPFD).

For nanoindentation experiments, *A. thaliana* WT (Col-0), *cesa3^je5^* (27), *PGX1-OE* (38), *pgx3-1*, and *PGX3-OE* (37) plants were used for measurements. Seedlings were grown at 23°C and relative humidity of 50–60%, under 16 h light/8 h dark cycles. Plants with emerging leaves #3 and #4 were selected for nanoindentation measurements on mature guard cells of their cotyledons. Plants were moved to a small petri dish filled with soil and a flat support at the center. Cotyledons were mounted on the support using Kwik-Cast silicone sealant on the abaxial side, so that the adaxial side was used for nanoindentation experiments. After mounting the samples, the soil in the petri dish was covered with plastic film to prevent dehydration. Plants were kept in light to induce stomatal opening, after which they were placed in the nanoindenter chamber.

### IIT (nanoindentation)

A Hysitron TI Premier Nanoindenter (Bruker, USA) was used to conduct nanoindentation experiments. The machine was equipped with a 50× objective so that guard cells could be easily identified. The diameter of the conical-type tip of the probe was ∼3 µm. A setpoint force of 2 µN was used to engage the tip with the middle of each targeted cell. Displacement control was set for the input load function, and loading, unloading, and lateral motions were set to 30 nm/s. During lateral indentation, the normal displacement was set to zero with respect to its previous position. Similarly, for normal indentation, the lateral displacement was set to zero so the probe would indent the center of the guard cell.

### Wall thickness measurements from TEM

Leaf samples of comparable age and position were cut into microcentrifuge tubes of fixative solution (2% glutaraldehyde and 2% paraformaldehyde in 0.1 M cacodylate buffer, pH 7.2) and fixed for 1 h at room temperature on a rocker. All samples were left in the same fixative overnight at 4°C and postfixed in 1% aqueous osmium tetroxide for 30 min, followed by a graded dehydration series in 50, 70, 90, 95 (×2), and 100% (×2) ethanol (15 min each). Spurr's resin was used as an embedding medium after solvent transition with ethanol and resin (50:50 ethanol:resin, followed by immersion twice in 100% resin for 2–3 h for each solution), and embedded samples were cured at 60–65°C for 24 h. Ultrathin sections (100 nm) were cut with a Leica UC7 ultramicrotome and stained with 2% uranyl acetate and Reynold's lead citrate. Images were acquired digitally with an AMT digital imaging system integrated with the Hitachi HT7800 TEM. TEM images with a resolution of 3.1 nm/pixel were analyzed to measure wall thickness at five positions.

### FE model of nanoindentation measurements

FE simulations of nanoindentation measurements were created for several cells of each genotype using commercial FE software (Abaqus, 2019) in order to estimate the wall moduli and turgor pressure. A Keyence laser microscope was used to measure stomatal complex length, complex width, and guard cell length and width for each model. The cross-sectional thickness distributions of guard cell walls were based on measurements from TEM. A model of each guard cell was individually constructed using the lofting method in SolidWorks software. The initial pore width used in the FE analyses was based on pore width at the closed state for Col-0 and mutants (17, 37, 38). Then, the structural model was imported into Abaqus. The conical indentation tip was scanned using the Keyence microscope, and its geometry was also imported into Abaqus. A model using a discrete coordinate system was assigned uniformly across the whole cell and was based on the orientations of cellulose and matrix polysaccharides in the guard cell wall (14, 15). The anisotropic behavior was assumed to be transversely isotropic with the symmetry axis defined by the cellulose in the direction of *E*_2_. Properties are defined by the plane transverse to the cellulose direction and by the plane that includes the cellulose direction. In this case, *E*_1_ = *E*_3_, and this value was assigned based on model iteration and convergence to the experimental results with an appropriate initial estimate (11). *E*_2_ defined the wall modulus along the circumferential direction of the cell, and this modulus was assumed larger at the start of the iterative modeling (14, 15, 59–61). Poisson's ratios were assumed as ν_12_ = ν_23_ = 0.3 and ν_13_ = 0.47. The shear moduli have the relation *G*_12_ = *G*_23_, and *G*_13_ has the constraint that *G*_13_ = *E*_1_/[2(1 + ν_13_)]. Little is known about the viscoelastic behavior of the wall parameters needed for the model. For this reason, all parameters were assumed to follow a Kelvin–Voigt model with a relaxation time of *τ* = 6.88 s (11, 40). For lateral indentation, contact properties such as the friction coefficient are expected to influence the force–displacement behavior which would impact the in-plane stiffness values of the wall that are needed to match the lateral measurements. For this reason, a series of experiments were used to determine the contact properties for the model. Lateral indentations starting from ±150 nm displacement from the center and increasing up to ±2 µm were performed on ∼10 cells along the longitudinal direction to investigate the contact properties between the guard cell and nanoindenter tip during lateral motion (see Fig. S5). These experiments revealed the point at which sliding began, the maximum lateral force, and the slope of the lateral force vs normal force (friction coefficient) during sliding. From these data, for all computational models, the friction coefficient, shear stress limit, and fraction of characteristic surface dimension were set to 0.3, 50 MPa, and 0.9, respectively. We performed two consecutive lateral indentations on a guard cell and compared the results to ensure that the cuticular wax layer was not removed by the tip during these experiments. Because no difference was observed, we concluded that no layer was removed by the probe during lateral motion (Fig. S5D). For boundary conditions, the material at the polar positions was confined, ventral edges were free of constraint, and dorsal edges were constrained in the vertical direction to represent constraints from adjacent pavement cells (11). The analysis was conducted in three steps: cell pressurization, normal nanoindentation, and lateral nanoindentation. The pore width at the end of the pressurization and the stiffness data at shallow (300 nm) and deep (1,250 nm) indentation depths were used iteratively with the model to determine the wall moduli and turgor pressure that provided the best match to the experiments.

### Statistical analysis

Statistical significance was determined using a Student’s t test with significance levels expressed using *P < 0.05, **P < 0.01, ***P < 0.001, ****P < 0.0001, and ns, not significant.

## Supplementary Material

pgad294_Supplementary_Data

## Data Availability

All data needed to evaluate the conclusions in the paper are present in the paper and/or the supplementary materials.
