# Supplementary material for: Stomatal opening efficiency is controlled by cell wall organization in Arabidopsis thaliana
Source: PNAS Nexus. 2023 Sep 11;2(9):pgad294. doi: 10.1093/pnasnexus/pgad294 (PMC10508357; doi:10.1093/pnasnexus/pgad294)
Supplement: pgad294_Supplementary_Data [file pgad294_supplementary_data.pdf]

Supplemental Material

Stomatal opening efficiency is controlled by cell wall organization in Arabidopsis thaliana

Sedighe Keynia, Leila Jaafar, You Zhou, Charles T. Anderson, Joseph A. Turner

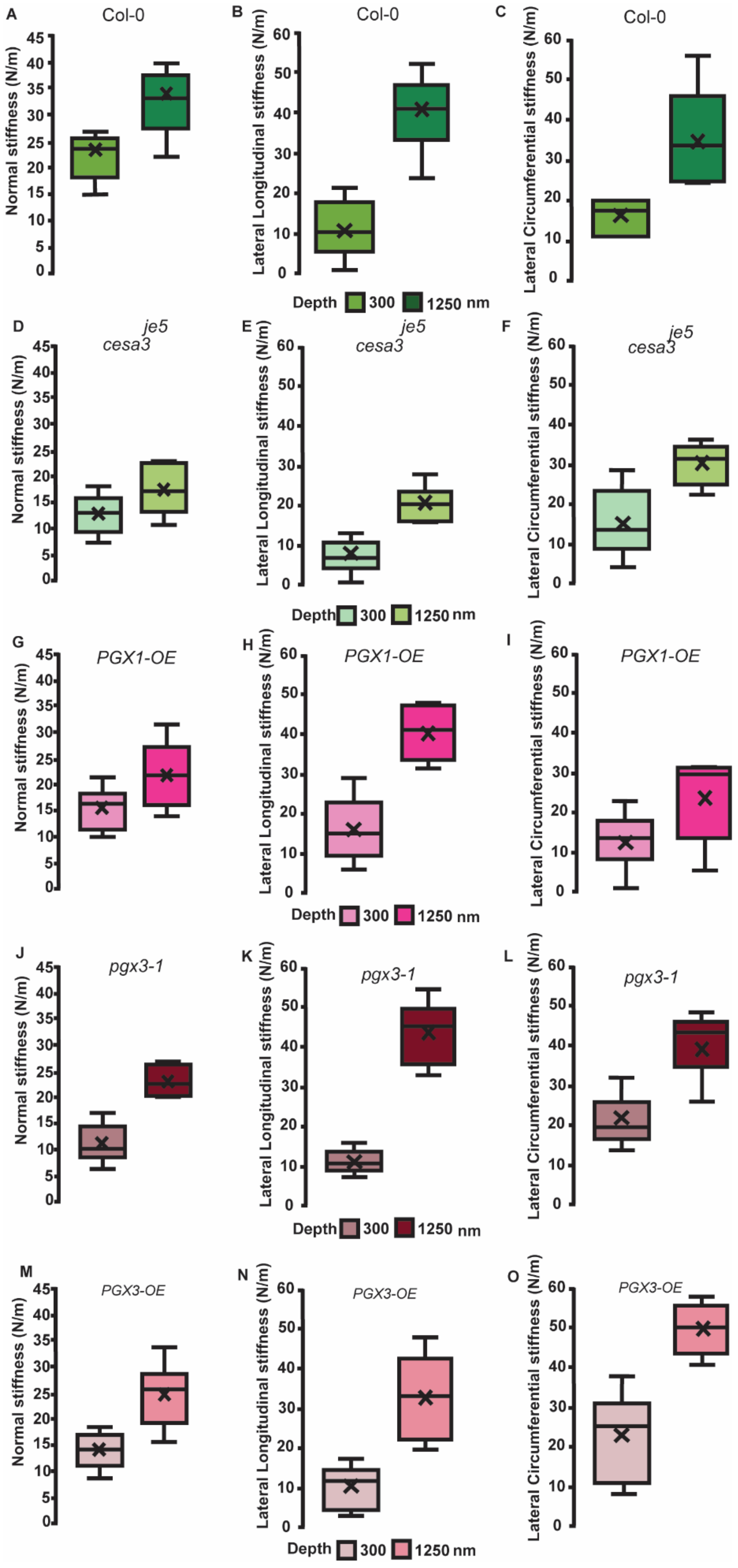

Fig. S1. Stiffness measurements of guard cell from different genotypes. Measured normal, longitudinal, and circumferential stiffnesses from shallow and deep indentation depths for Col-0 (**A**, **B** and **C**), *cesa3*^{je5} (**D**, **E** and **F**), *pgx1-OE* (**G**, **H** and **I**), *pgx3-1* (**J**, **K** and **L**), and *PGX3-OE* (**M**, **N**, and **O**). (related to Figure 1)

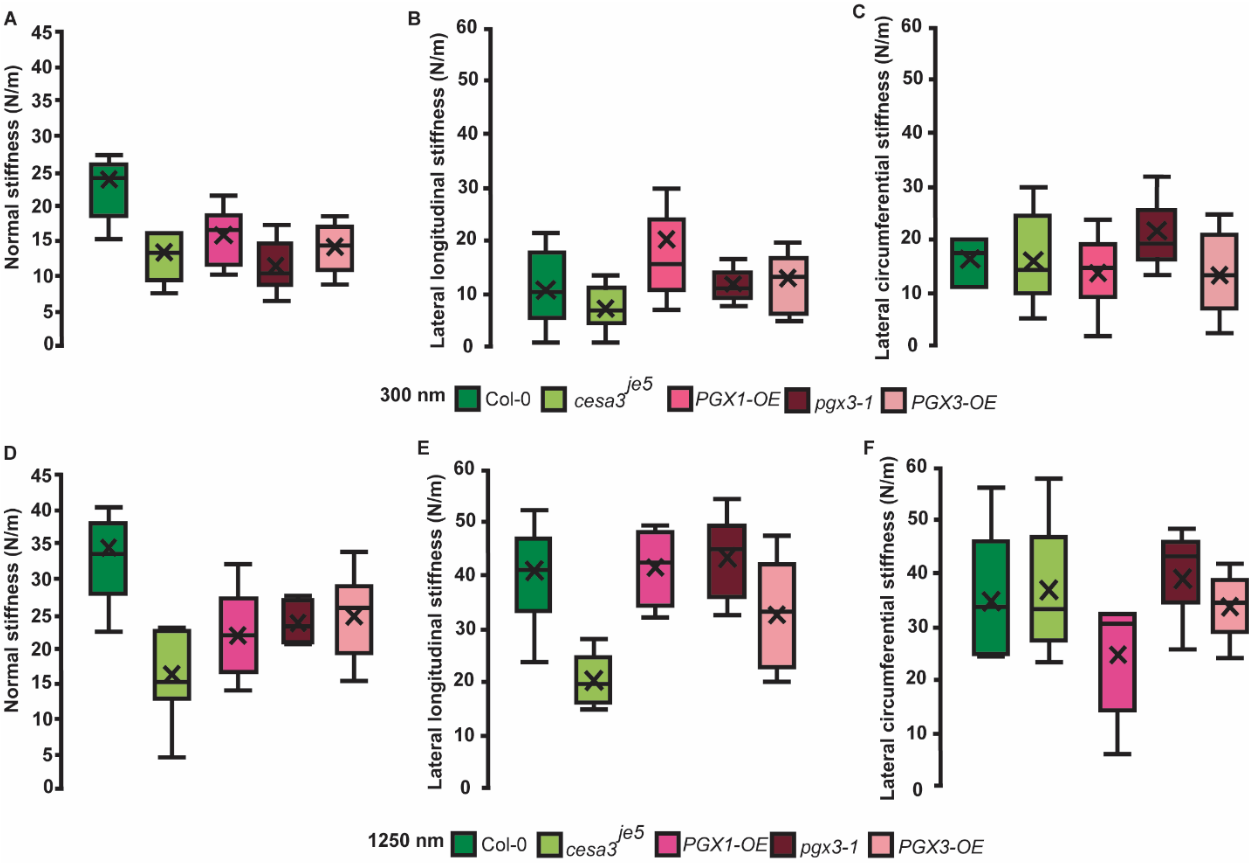

Fig. S2. Stiffness measurements of Col-0 and mutants guard cells. Normal, longitudinal, and circumferential stiffness of the mature guard cells for Col-0, *cesa*^{je5}, *PGX1-OE*, *pgx3-1* and *PGX 3-OE* at shallow (**A**, **B** and **C**) and deep depths (**D**, **E** and **F**) for more than 10 guard cells each. (related to Figure 1)

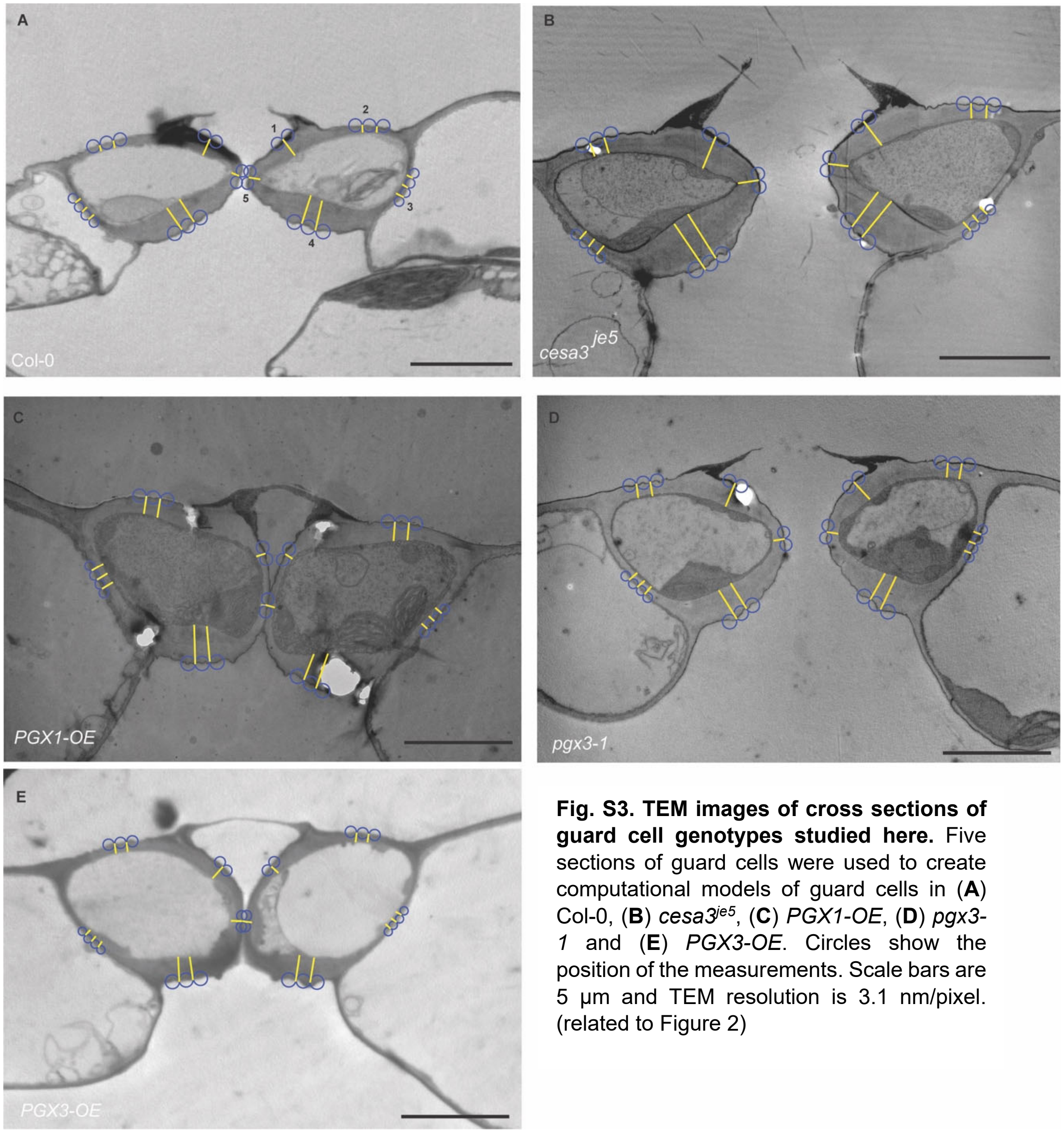

Fig. S3. TEM images of cross sections of guard cell genotypes studied here. Five sections of guard cells were used to create computational models of guard cells in (**A**) Col-0, (**B**) *cesa3*^{je5}, (**C**) *PGX1-OE*, (**D**) *pgx3-1* and (**E**) *PGX3-OE*. Circles show the position of the measurements. Scale bars are 5 µm and TEM resolution is 3.1 nm/pixel. (related to Figure 2)

Table S1. Cell wall thickness information in µm based on three measurements at specific locations. (related to Figure 2)

Genotype	Outer wall at cuticular ledge	Outer wall away cuticular ledge	Dorsal wall	Inner wall	Ventral wall
**Col-0**	1.27 ± 0.15	0.72 ± 0.06	0.64 ± 0.12	1.87 ± 0.29	0.76 ± 0.07
***cesa3*^{je5}**	1.34 ± 0.08	0.74 ± 0.04	0.32 ± 0.07	2.39 ± 0.28	0.94 ± 0.21
PGX1-OE	1.18 ± 0.14	0.64 ± 0.10	0.36 ± 0.04	1.72 ± 0.03	0.40 ± 0.24
pgx3-1	1.06 ± 0.11	0.66 ± 0.02	0.33 ± 0.03	1.65 ± 0.06	0.56 ± 0.14
PGX3-OE	0.74 ± 0.12	0.48 ± 0.19	0.32 ± 0.07	1.27 ± 0.42	0.25 ± 0.04

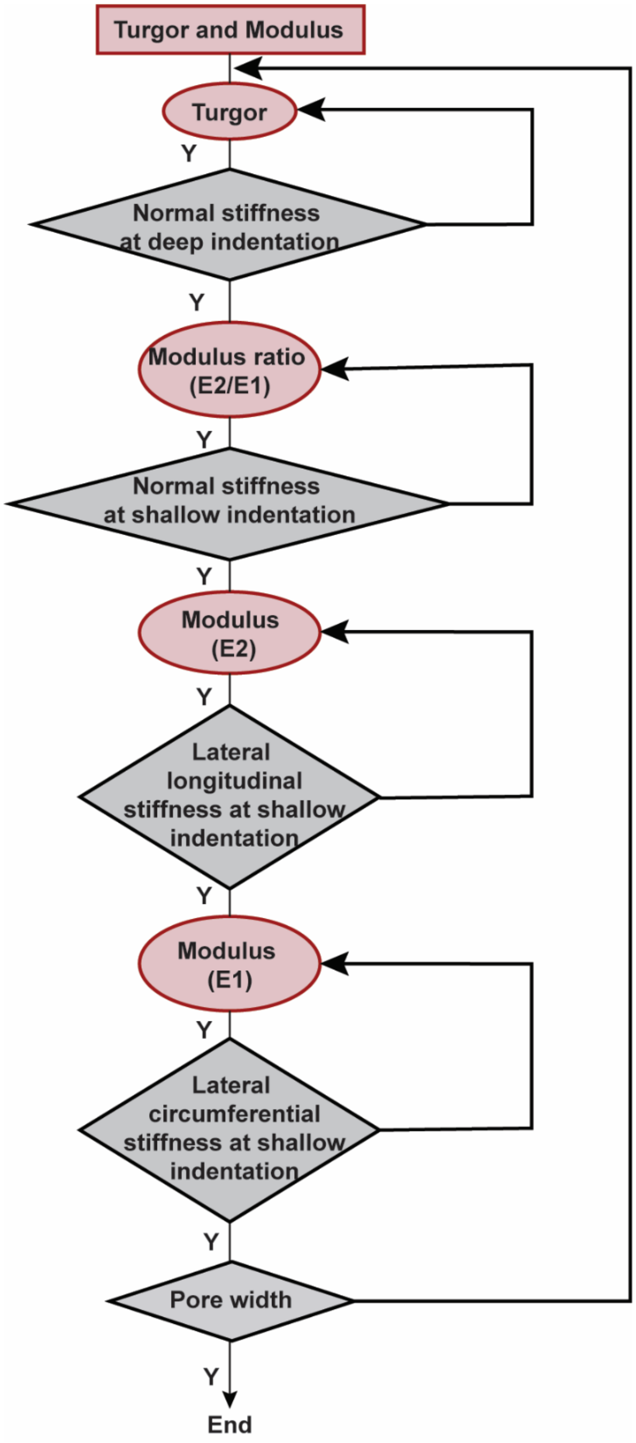

Fig. S4. Iteration Flowchart. The flowchart of the iteration process for validating mechanical properties of the cell wall assigned to the FEM based on three orthogonal nanoindentation measurements and pore width. (related to Figure 3)

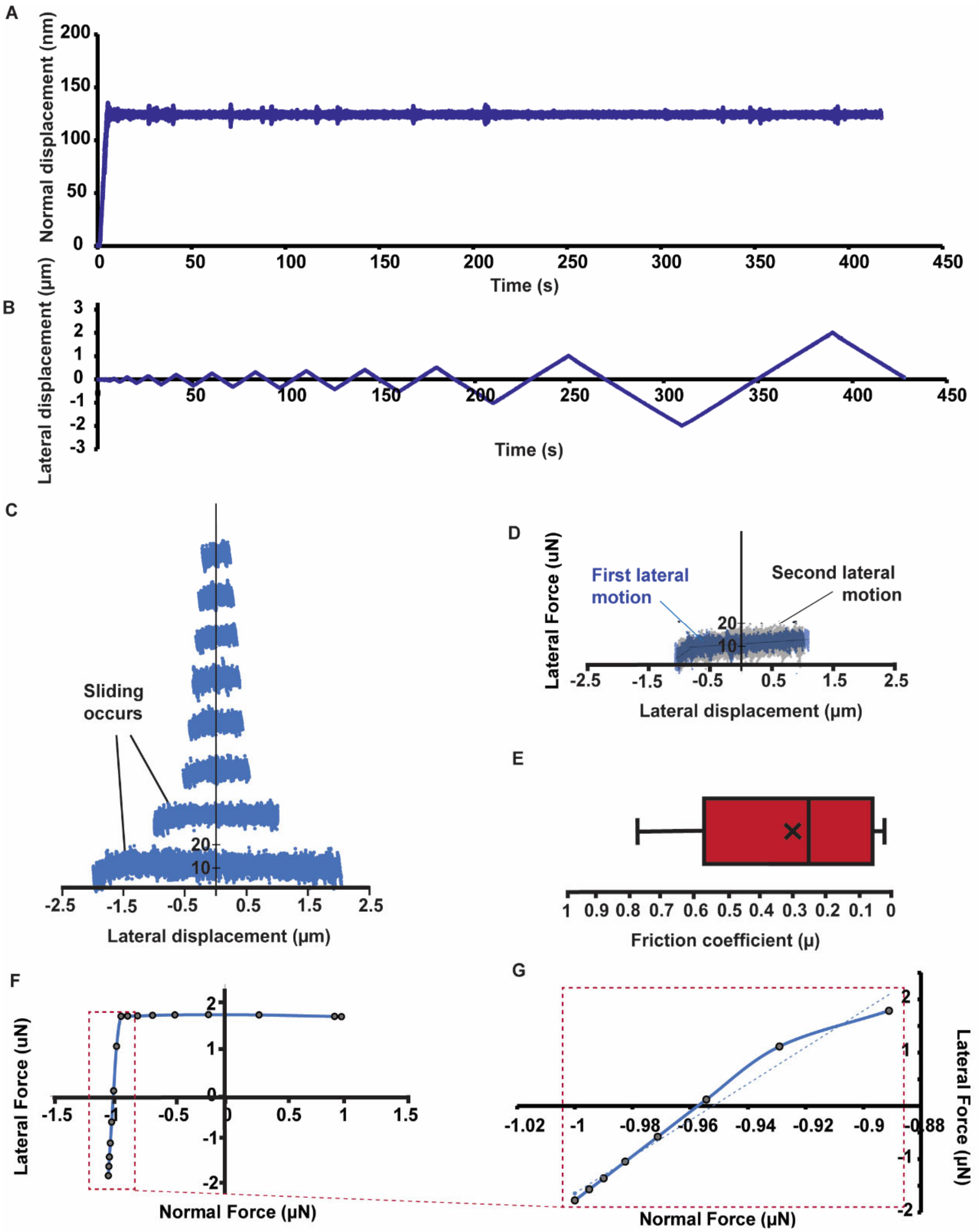

Fig. S5. Determining the contact properties between the tip and guard cell wall. (**A**) Normal, and (**B**) Lateral indentation displacement profile. The tip indents to a normal depth of 150 nm and then the lateral indentation begins with small displacements and increases to the maximum lateral displacement (5 µm). The result of indentation is shown in (**C)** which shows the lateral force vs lateral displacement for each set of lateral measurement (starting from the center of guard cell and ending at the same location). A 20 µN offset is used for each result for better visual display. (**D**) An identical result of repeated lateral indentation shows that no layer is removed after lateral indentation. (**E**) Slope of the sliding sections of the lateral force vs displacement measurements of 10 cells in the longitudinal direction gives the friction coefficient with mean of ~0.3. (**F,G**) The maximum normal force before sliding and the maximum lateral force provides allows the other parameters of the contact to be defined in the model. (related to Figure 3)

Table S2. Mechanical properties determined from the FEM iteration process to match the nanoindentation apparent stiffness data. A different number of results were obtained for each genotype due to differences in available experimental data. (related to Figure 3)

Genotype	E_1 (Longitudinal)	E_2 (Circumferential)	Ratio (E_2/E_1)	$G_{12} = G_{32}$ (MPa)	G_{13} (MPa)	ΔP (MPa)
Col-0	37.8	94.1	2.49	37.8	12.9	0.79
	44.5	94.1	2.12	44.5	15.1	0.60
	62.5	135.2	2.16	62.5	21.3	1.10
	37.8	94.1	2.49	37.8	12.9	0.90
	30.0	85.0	2.83	30.0	10.2	1.00
	39.4	97.0	2.46	39.4	13.4	0.85
	20.4	76.4	3.75	20.5	6.94	0.50
	13.5	64.7	4.79	13.5	4.59	0.65
	17.3	70.6	4.08	17.3	5.89	0.60
cesa3^{je5}	40.3	67.0	1.66	40.3	13.7	0.85
	40.3	88.2	2.19	40.3	13.7	0.74
	29.4	35.3	1.20	29.4	10.0	0.72
	23.3	41.2	1.77	23.3	7.93	0.60
PGX1-OE	30.1	81.0	2.69	30.1	10.2	0.92
	10.0	35.0	3.50	10.0	3.40	0.64
	20.2	70.4	3.48	20.2	6.88	0.68
	35.3	85.3	2.42	35.3	12.0	0.67
	30.1	87.0	2.89	30.1	10.2	0.83
	30.1	87.0	2.89	30.1	10.2	0.83
	37.6	89.4	2.38	37.6	12.8	0.94
	37.6	89.4	2.38	37.6	12.8	1.00
	37.6	89.4	2.38	37.6	12.8	1.00
pgx3-1	85.8	147.0	1.71	85.8	29.2	0.80
	87.4	158.8	1.82	87.4	29.7	1.20
	58.8	100.0	1.70	58.8	20.0	0.60
	71.0	147.0	2.07	71.0	24.1	0.72
	99.6	147.0	1.48	99.6	33.9	0.68
	136.3	205.8	1.51	136.3	46.4	1.20
PGX3-OE	42.5	105.8	2.49	42.5	14.5	1.50
	40.0	89.3	2.23	40.0	13.6	0.76
	37.6	89.4	2.38	37.6	12.8	1.00
	40.0	89.4	2.23	40.0	13.6	1.00
	40.0	89.3	2.23	40.0	13.6	1.00
	37.6	89.4	2.38	37.6	12.8	1.00
	70.0	147.0	2.10	70.0	23.8	1.20
	37.6	89.4	2.38	37.6	12.8	1.00

*$\nu_{13} = 0.47$ and $\nu_{23} = \nu_{21} = 0.3$
